# Robotic nanoparticle delivery systems for tumor hyperthermia: clinical readiness and challenges

**DOI:** 10.1097/MS9.0000000000004401

**Published:** 2025-11-22

**Authors:** Hasibullah Aminpoor, Muhammad Khizar, Muhammad Zaib, Sharjeel Haider, Hasiba Karimi

**Affiliations:** a Faculty of Medicine, Kabul University of Medical Sciences “Abu Ali Ibn Sina”, Kabul, Afghanistan; b Faculty of Medicine, Georgian American University, Manifest Medical Research Co.Tbilisi, Georgia; c Faculty of Medicine, FMH College of Medicine and Dentistry, Lahore, Pakistan; d Faculty of Medicine, Bezmialem Vakif University, Istanbul, Turkey

**Keywords:** cancer therapy, nanoparticles, robotic delivery, tumor hyperthermia

## Abstract

Robotic nanoparticle delivery systems for tumor hyperthermia are redefining precision oncology. By integrating magnetic nanoparticles, robotics, and real-time imaging, these systems enable controlled heating of malignant tissue while sparing adjacent structures. Magnetic nanoparticle hyperthermia employs iron oxide nanoparticles activated by alternating magnetic fields to generate localized heat. Advances in Korea, Germany, and China demonstrate the effectiveness of microrobots and magnetic swarms in enhancing nanoparticle targeting and uniform thermal deposition. Image-guided robotic systems with MRI and ultrasound thermometry further optimize safety and reproducibility. Clinically, Germany’s MagForce Nanotherm® device is approved for glioblastoma, with ongoing international trials in prostate and pancreatic cancers. However, consistent dosing, nanoparticle biosafety, and standardized protocols remain pressing challenges. With cross-disciplinary collaboration, robotic hyperthermia could soon become a minimally invasive, targeted option for complex solid tumors. This emerging field underscores the need for ethical transparency and adherence to reporting standards for artificial intelligence in medical technology development.

Robotic nanoparticle delivery systems represent a promising evolution in oncologic hyperthermia. Tumor hyperthermia, achieved through localized heating of malignant tissue, enhances tumor susceptibility to chemotherapy and radiotherapy. This letter aims to review recent advances in robotic nanoparticle delivery for tumor hyperthermia, identify current challenges in clinical translation, and discuss emerging strategies to improve targeting precision and safety.

Magnetic nanoparticle hyperthermia (MNH) utilizes iron oxide nanoparticles activated by alternating magnetic fields to induce heat specifically within tumor microenvironments^[1]^. Yet only a fraction of injected nanoparticles reaches solid tumors, necessitating innovations in guided delivery and targeting precision^[2]^.

Recent developments in magnetically controlled microrobotics have transformed this field. In Korea, Park *et al* fabricated biodegradable Fe₃O₄ microrobots capable of drug release and localized heating under magnetic control^[3]^. In Germany, Medina-Sánchez and colleagues engineered magnetic nanoparticle swarms that actively navigate to glioblastoma cells, achieving localized cytotoxicity^[4]^. Gold nanoshells and carbon-based nanoparticles have similarly been applied for photothermal ablation in breast and pancreatic cancers^[1]^. Such multimodal systems, integrating magnetic guidance and surface functionalization, significantly enhance heat localization and biocompatibility.

Robotic guidance technologies are now enabling real-time precision. Tonthat *et al* developed an automated coil-and-robotic-arm system achieving millimeter-level accuracy in magnetic heat delivery^[5]^. Integration with MRI and ultrasound thermometry permits live mapping of thermal gradients, improving safety and reproducibility. Preclinical studies in China and the United States show that static magnetic targeting can increase nanoparticle tumor deposition up to five-fold compared to passive administration^[2]^, demonstrating the potential for enhanced delivery efficiency.

Despite these advances, several challenges remain. Field penetration depth limits effective hyperthermia in deep-seated tumors, as heating efficiency decreases with distance from the magnetic field source. Dosimetry standardization is lacking, with no universally accepted protocols for measuring thermal dose or assessing tissue response during MNH^[1]^. Long-term biocompatibility of nanoparticles also requires further investigation; iron oxide particles can accumulate in the liver and spleen, and their clearance kinetics and immune interactions are not fully understood. Addressing these issues is essential for safe clinical translation.

Clinical adoption is progressing. Germany’s MagForce Nanotherm® system has gained approval for recurrent glioblastoma, while U.S. and European trials are evaluating similar devices in prostate and pancreatic malignancies^[4]^.

In conclusion, robotic nanoparticle hyperthermia marks a significant step toward precision, image-guided oncologic intervention. Integrating robotic navigation with functional nanomaterials and imaging feedback may allow safe, minimally invasive tumor ablation. Future research should emphasize nanoparticle biocompatibility, rigorous clinical validation, and harmonized regulatory frameworks. Our work aligns with the TITAN Guidelines emphasizing transparency in AI-assisted healthcare interventions^[6]^.

## Data Availability

Not applicable.
